# Hot Ground State Cooling Following Ultrafast Photoisomerization: Time-Resolved Infrared Spectroscopy

**DOI:** 10.1021/acs.jpcb.5c07581

**Published:** 2025-12-10

**Authors:** James N. Bull, Mark H. Stockett, Pratip Chakraborty, Eleanor K. Ashworth, Anam Fatima, Vincent J. Esposito, Gregory M. Greetham, Partha Malakar, Stephen R. Meech

**Affiliations:** † Chemistry, Faculty of Science, 6106 University of East Anglia, Norwich NR4 7TJ, U.K.; ‡ Department of Physics, 7675 Stockholm University, SE-10691 Stockholm, Sweden; § Chemistry, Schmid College of Science and Technology, Chapman University, Orange, California 92866, United States; ∥ Central Laser Facility, Research Complex at Harwell, Rutherford Appleton Laboratory, Didcot OX11 0QX, U.K.

## Abstract

The ultrafast photophysics of many isomerizing molecules involves subpicosecond formation of a twisted hot ground state, which transfers energy to the environment through vibrational relaxation (cooling) over several picoseconds. In time-resolved infrared (TR-IR) spectroscopy, hot ground state transients show frequency shifts and band reshapings, which cannot be described through kinetic models that assume static spectral functions. We report a simple anharmonic cascade framework, which uses a single adjustable parameter associated with scaling the probability of vibrational energy transfer to the environment, for describing hot ground state cooling (HGSC) in TR-IR spectroscopy. The model is demonstrated against measurements on the cyan fluorescent protein chromophore. To best describe HGSC band shape evolution, the model utilizes *ab initio* data on anharmonic vibrational structure and nonadiabatic molecular dynamics trajectories of S_1_→ S_0_ internal conversion for realistic vibration occupation numbers of the nascent hot ground state. The modeling framework is readily extended to include mode-specific rates for intermolecular energy transfer and can be applied to any ultrafast isomerizing molecule for which anharmonic vibrational properties can be computed.

## Graphical abstract

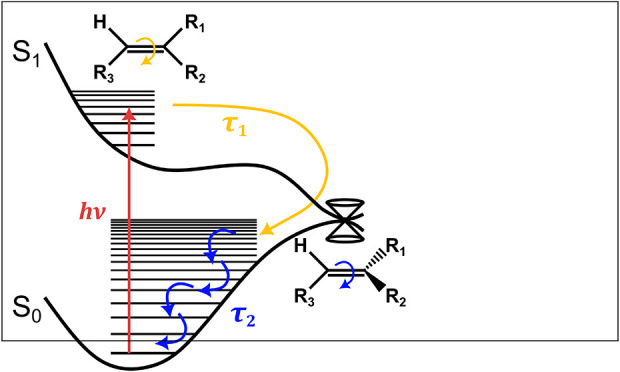

## Introduction

Ultrafast photoisomerization or molecular rotor motion of a double bond in a chromophore often occurs via passage through a conical intersection seam connecting the ground and first electronically excited state of the same spin multiplicity.^1^ This is a widespread phenomenon, occurring throughout nature in mammalian vision^2^ and photoactive proteins,^3,4^ in phototactic responses in light-sensitive bacteria,^5,6^ and in technological applications such as molecular photoswitches^7^ and photomolecular motors.^8–11^ Because *E*-*Z* (*trans–cis*) photoisomerizations usually involve substantial twisting about a double bond (azobenzenes may isomerize through inversions^12^), vibrational modes associated with the displacement become highly energized on formation of the twisted ground electronic state. In turn, it takes some picoseconds for all of intramolecular vibrational energy redistribution (IVR), intermolecular vibrational energy transfer (IET) to the environment, and geometric relaxation – collectively termed hot ground state cooling (HGSC) – to occur. In experiments based on ultrafast vibrational spectroscopy, HGSC transients contain averaged fingerprints of photoisomerization pathways. Notably, in time-resolved infrared (TR-IR) spectroscopy, HGSC signatures can be observed for energized vibrational modes, with their evolution linked to the nascent hot ground state geometries and nonequilibrium vibrational energy distribution (Figure 1). In ultrafast TR-IR, because HGSC for the highly energized vibrational modes manifest as an evolving spectral profile with time (reshaping toward higher wavenumber), global fit models that assume static spectral basis functions are unable to provide physically meaningful kinetics. This work develops a simple framework for predicting HGSC active modes and corresponding band evolution using data on internal conversion from nonadiabatic molecular dynamics trajectories, with the purpose of helping to interpret experimental TR-IR data.

**Figure 1:**
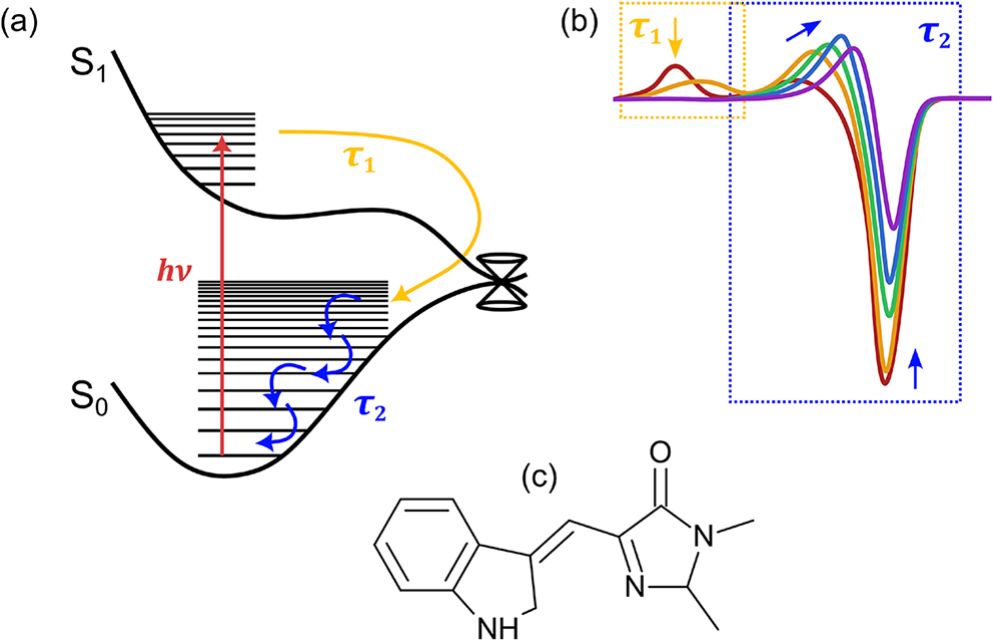
Hot ground state cooling (HGSC) dynamics in a photoisomerizing molecule involving S_0_ and S_1_ electronic states: (a) Potential energy surfaces for an ultrafast photoisomerization. Lifetime τ_1_ is linked with loss of the S_1_ state via passage through an isomerizing conical intersection seam, and lifetime τ_2_ corresponds to HGSC. (b) Manifestation of these dynamics in ultrafast TR-IR spectra. The spectral shape and time-dependent reshaping of the HGSC profile for a given vibrational mode is defined by anharmonicity coefficients, the vibrational energy distribution, and vibrational energy relaxation processes. Note that the integrated ground-state bleach over a given band may be larger than the corresponding hot ground state absorption. (c) Cyan fluorescent protein chromophore (shown as the *Z*1 isomer).

The relaxation of vibrational energy from a nascent hot ground state molecule in solution subsumes two processes:^13,14^ (i) IVR, where a nonequilibrium distribution of vibrational energy becomes statistically redistributed among internal modes, and (ii) IET, where vibrational energy is dissipated into individual and collective vibrations of the solvent molecules. Low frequency modes are typically more efficient for IET, requiring IVR to accesses such modes that, in turn, efficiently transfer energy to solvent; these dynamics lead to complex solute- and solvent-dependent IET rates.^15–18^ The rates of IVR and IET have been well reviewed for small molecules (a few atoms) as they provide the clearest opportunities to elucidate microscopic analysis.^13,15,19^ For aromatic and conjugated molecules with 20 or more atoms and a spread of vibrational frequencies, IVR rates are usually a few picoseconds,^20–22^ such as for photoisomerizing *Z*-stilbene (sub-300 fs internal conversion) with a high degree of vibrational excitation over the mid-IR range.^23–27^ The implication is that IET and IVR occur on similar time scales need to be considered in concert and a HGSC model for ultrafast dynamics requires nonequilibrium vibration occupation numbers.

Preliminary foundations for describing HGSC have been considered by Shelby et al.^28^ and Hamm et al.,^29^ with the latter arguing the importance of an anharmonic approach. The limiting cases of ultrafast IVR, i.e., HGSC occurs from an equilibrated hot molecule (termed the rapid exchange limit in this work), and slow IVR with selective excitation of specific modes, were discussed in the latter. In other work, Murdock et al.^30^ used first-order kinetics in a harmonic approximation to fit sigmoidal-like functions to HGSC bleach amplitudes in TR-IR spectra of thiophene carbonyl stretch modes.

We report a trajectory-based anharmonic cascade framework for describing HGSC in ultrafast photoisomerizing molecules, illustrated using the cyan fluorescent protein chromophore (Figure 1c, denoted cyan) dissolved in CD_3_OD (deuterated methanol). Application of the HGSC framework requires input data on the (anharmonic) vibrations and expected vibration occupation numbers for the nascent hot ground state. The cyan chromophore is well suited for such a demonstration because: (i) The excited-state (S_1_) lifetime is <500 fs, as determined by transient absorption and femtosecond fluorescence upconversion spectroscopies,^31^ with most electronic energy rapidly converted into vibrational energy. (ii) There is a single internal conversion pathway involving *Z*–*E* twisting about a methylene double bond with a well-defined conical intersection seam with a photoisomerization quantum yield of a few percent in solution (meaning that analysis need consider only the synthesized *Z* isomer). (iii) Cyan is readily studied using ultrafast TR-IR spectroscopy in CD_3_OD.^31^ While it is not possible to use such modeling to extract detailed anharmonic information from experimental TR-IR spectra, the model is useful for determining which modes might be active or displaced versus spectators in a photoisomerization mechanism, and for predicting HGSC band evolution in order to help interpret TR-IR spectra. For example, to help distinguish excited state absorption from HGSC in complex TR-IR spectra; this situation is particularly relevant for cyan and related fluorescent protein chromophores due to potential ambiguities in how many electronic excited states are involved in the ultrafast photophysics.^31^

## Methods

### HGSC Model Framework

Our HGSC framework considers the total energy *E*, relative to the zero point energy, of a vibrational configuration **n** = (*n*_1_, *n*_2_,..., *n*_*N*_), where *N* is the number of vibrational modes and *n*_*i*_ is the occupation number (quantum number) of mode *i*, in second-order vibrational perturbation theory (VPT2) as being given by the Dunham expansion:^32^

1 $$E=\sum_{i}h\nu_{i}\left(n_{i}+\frac{1}{2}\right)+\sum_{i\leq j}X_{\mathrm{ij}}\left(n_{i}+\frac{1}{2}\right)\left(n_{j}+\frac{1}{2}\right)$$

where ν_*i*_ is the frequency of mode *i*, and *X*_*ij*_ are elements of the anharmonic **X** matrix (taken from electronic structure calculations described in the next section). The **X** matrix contains anharmonic coupling constants between the fundamental vibrational modes, with diagonal terms describing self-anharmonicities and off-diagonal terms describing mode–mode couplings.

The fundamental band transition energies are

2 $$\Delta E_{fund,i}=h\nu_{i}+X_{\mathrm{ii}}\left(n_{i}+\frac{3}{2}\right)+\sum_{\mathrm{ij}}X_{\mathrm{ij}}\left(n_{j}+\frac{1}{2}\right)$$

Similar expressions for the first overtones and 1 + 1 combination bands are given in the Supporting Information. Note that we prefer *n*_*i*_ as a occupation (quantum) number rather than conventional *v*_*i*_ in order to clearly distinguish from mode frequency, ν_*i*_. The IR intensity of a fundamental band is

3 $$I_{i}=I_{i}^{0}\left(n_{i}+1\right)\left(\frac{\Delta E_{\mathrm{fund},i}}{\Delta E_{\mathrm{fund},i}^{0}}\right)$$

where the superscript 0 indicates the transition energy and intensity when *n*_*i*_ = 0 for all *i* (i.e., the default anharmonic values from a Gaussian VPT2 calculation). The scaling of the IR intensity with *n*_*i*_ + 1 is strictly valid for only the harmonic oscillator. Similar expressions for the first overtones and 1 + 1 combination bands are given in the Supporting Information.

The first step in obtaining the simulated IR spectrum at *T* = 300 K utilized a multicanonical (MUCA) sampling algorithm for the space of vibrational occupation numbers (i.e., giving the thermal occupation of each vibrational mode *n*_*i*_ at room temperature).^33,34^ The total energy range of interest (≈ 1 eV) in the IR spectrum was divided into four nonoverlapping windows. For each window, a histogram **H** with bin widths of 16 cm^–1^ was first set to zeros, as was a spectrum matrix with a row (spectrum) for each energy bin. Starting from the random sampled configuration **n**, a new configuration **n**_prop_ is proposed in which each *n*_*i*_ may decrease or increase by one quantum, subject to *n*_*i*_ ≥ 0. The probability of proposing a downward step is $p_{\mathrm{down}}=\frac{1}{N}$ , while that of an upward step is $p_{\mathrm{up}}=\frac{r}{N}$ , where *r* = 1.08 was found to accelerate convergence. The proposed configuration was first validated by ensuring that the total energy (eq 1) remained in the window and that all fundamental transition energies obeyed Δ*E*_fund_ > 0. Valid configurations were accepted with a probability given by a Metropolis-Hastings condition:^35^

4 $$p_{\mathrm{acc}}=\min\left[1,\frac{\rho \left(E\right)}{\rho \left(E_{\mathrm{prop}}\right)}r\Delta n\right]$$

where *E* and *E*_prop_ are the total energy of the current and proposed configurations, ρ­(*E*) is the density of vibrational states, and Δ*n* is the difference in the total number of quanta between the current and proposed configurations. Here, the ρ­(*E*) was calculated using the Stein-Rabinovitch exact state counting algorithm,^36^ treating the vibrations as separable anharmonic oscillators i.e., assuming *X*_*i*≠*j*_ = 0. We note that published computations of fully coupled level densities using the Wang–Landau algorithm^37^ have insignificant differences from uncoupled anharmonic calculations for *E* < 1 eV. If the proposed configuration is accepted, **n**_prop_ replaces **n** and *E*_prop_ replaces *E*. Regardless of acceptance, the energy bin of **H** corresponding to *E* is incremented by 1. The transition energies and IR intensities for the fundamental, first overtone, and 1 + 1 combination bands are computed for **n** and the resulting spectrum is added to the row of the spectral matrix corresponding to the same energy bin. New steps are proposed until a flatness condition is met

5 $$\mathrm{all}\left[\frac{H-\left\langle H\right\rangle }{\left\langle H\right\rangle }<\alpha \right]$$

where ⟨**H**⟩ is the mean value of **H**, and α = 0.25. Each row *i* of the spectral matrix is divided by the number of samples in the corresponding energy bin *H*_*i*_. The final matrix is assembled from the four energy windows, each of which was sampled by six independent walks with different random initial configurations. This matrix contains the absorption spectrum associated with the microcanonical energy *E* corresponding to each row. To obtain the IR spectrum for a canonical temperature *T*, a weighted sum of the rows is performed, with the weights given by the Boltzmann distribution for that temperature.

Time-dependent IR spectra were simulated with a kinetic Monte Carlo approach that uses similar logic to the MUCA simulation. For cyan, two sets of simulations were performed: (i) projected vibration occupation numbers from nonadiabatic molecular dynamics (NAMD) trajectories, determined from surface-hopping geometries at the S_1_→ S_0_ conical intersection seam (described in the NAMD Trajectories section), as the initial configurations; (ii) a set of random configurations yielding a similar distribution of initial total energies was generated – this corresponds to the rapid exchange limit for which IVR fully precedes IET. To each occupancy configuration, a randomly sampled configuration from a *T* = 300 K distribution **n**_*T*_ was added to include the initial temperature of the system prior to excitation, giving initial configuration **n**. The Monte Carlo trajectories evolved as follows. For each **n**, a blank spectral matrix was initialized with a row for each time step (*dt* = 5 fs). The probability of proposing a downward step in *n*_*i*_ is *p*_down*, i*_ = *qn*_*i*_ν_*i*_ *dt*, where *q* = 0.1 is an arbitrary scaling factor adjusted so that the time-evolution of the spectrum roughly matches that of the experiment – this is the single adjustable parameter in our HGSC model that affects only the rate, not the transient spectral shape. The probability of proposing an upward step, corresponding to energy gained from the bath, is *p*_up,*i*_ = *q*(*n*_*i*_ + 1)­ν_*i*_ *dt* · e^–*hν*/*k*_B_ *T*^, where *T* = 300 K is the temperature of the bath. We assume the isothermal bath approximation, where energy dissipation within the bath is sufficiently rapid so that the local bath temperature (first solvation shell of the molecule) is the same as the bulk bath temperature. The acceptance probability is *p*_acc_ = e^–(*E*–*E*_prop_)/*k*_B_ *T*^. Unlike in the MUCA strategy above, configurations were not required to be within the valid range of the Dunham expression because some of the initial configurations given from NAMD projections are outside of these bounds and would never evolve. Negative transition energies are assigned zero intensity. Regardless of acceptance, the transition energies and IR intensities are calculated and stored in the row of the spectral matrix corresponding to the current time step. Many trajectories (576) were run for each initial configuration, and were averaged to construct the total time-dependent spectral matrix (from 46656 points).

### Anharmonic Vibrational Properties

The spectral evolution and associated intensity changes of HGSC transients in TR-IR are largely governed by anharmonicity effects of highly excited vibrational modes, necessitating incorporation of fundamental bands, 1 + 1 combination bands, first overtones, anharmonicity constants for each mode (e.g., **X** matrix). For cyan, the anharmonic IR properties were computed via a quartic force field (QFF), which is a truncated fourth-order Taylor expansion of the potential portion of the Watson Hamiltonian, given by

6 $$V=\frac{1}{2}\sum_{i,j}^{3N}\left(\frac{\partial^{2}V}{\partial X_{i}\partial X_{j}}\right)X_{i}X_{j}+\frac{1}{6}\sum_{i,j,k}^{3N}\left(\frac{\partial^{3}V}{\partial X_{i}\partial X_{j}\partial X_{k}}\right)X_{i}X_{j}X_{k}+\frac{1}{24}\sum_{i,j,k,l}^{3N}\left(\frac{\partial^{4}V}{\partial X_{i}\partial X_{j}\partial X_{k}\partial X_{l}}\right)X_{i}X_{j}X_{k}X_{l}$$

where *X* is a nuclear displacement. Optimized geometries, fundamental modes, and harmonic frequencies were determined at the B3LYP/N07D level of theory^38,39^ with Gaussian 16.C01.^40^ These computations were performed using very tight optimization criteria (1 × 10^–12^) and a custom integration grid consisting of 200 radial shells and 974 angular points per shell. The N07D basis set takes 6–31G and adds selected dispersion and polarization functions that improve the accuracy of calculated vibrational frequencies for large aromatic and highly conjugated molecules.^41^ Next, the normal coordinate QFF (quadratic, cubic, and semidiagonal quartic force constants) was computed using small displacements (0.01 Å steps) along all normal coordinates, with a linear transformation providing a Cartesian coordinate QFF.^42^ The vibrational spectrum was computed using second order vibrational perturbation theory (VPT2)^43–45^ within the software program Spectro.^46^ The VPT2 method implemented in Spectro utilizes a resonance polyad matrix approach.^47–49^ When two vibrational states of the same symmetry are close in frequency, they create a near-singularity in the conventional VPT2 equation. Here, the interacting states were removed from the VPT2 computation and were included in resonance polyad matrices based on symmetry. These matrices allow for the treatment of resonance effects while also accounting for states that simultaneously participate in multiple resonance interactions, termed resonance chaining. Additionally, the resonance polyads treat the redistribution of intensity between coupled states by using the eigenvectors of the diagonalized matrix.^49,50^ The maximum frequency separation for a resonance in the polyad treatment was set to 200 cm^–1^.^49,51^ Our anharmonic computation strategy with Spectro typically produces (gas phase) frequencies with a mean absolute error between theory and gas-phase experiments of ≈ 5 cm^–1^, and shows good agreement with (gas phase) experimental intensities – see refs 52,53 , and references therein; harmonic approximation computations have poor agreement with experiment. While B3LYP is suitable for describing ground electronic states of main group organic at equilibrium geometries, it has well-known failures for excited states and particularly those with charge-transfer character or for excited-state geometries in the vicinity of a conical intersection.^54^ Consequently, we determined harmonic and anharmonic frequencies using the BH&HLYP/N07D level of theory (where BH&HLYP has 50% Hartree–Fock exchange).^38^ Harmonic frequencies were checked against MRSF-TDDFT values (described next) and were found to have little deviation.

### NAMD Trajectories

NAMD dynamics on cyan were performed using MRSF-TDDFT^55^ at the BH&HLYP/6–31G*level of theory^56^ in GAMESS-US (July 2024 R2 release).^57^ The NAMD trajectories used Tully’s fewest-switches surface-hopping algorithm,^58^ with nonadiabatic coupling vectors computed numerically using a fast overlap method.^59,60^ Velocity Verlet was used for integration. The trajectories were propagated for 1500 fs (1.5 ps) with a time step of 0.5 fs. Energy conservation during the hops was ensured by rescaling of the velocities. The nuclear degrees of freedom were propagated with a subtime-step size of 10^–5^ fs for the electronic degrees of freedom.^61,62^ No corrections for decoherence were applied. 110 trajectories were initiated on the S_1_ state and 97 were included in the statistics (11 removed due to SCF convergence issues and backward hops because decoherence was not considered). We note that the same MRSF-TDDFT methodology has been benchmarked against CASSCF and XMS-CASPT2 potential energy surfaces for the related green fluorescent protein chromophore.^62,63^ The initial occupation numbers *n*_*i*_ after surface hopping were computed for each trajectory by projecting the equilibrium and surface hopping geometries with the (mass weighted) normal vectors for the equilibrium geometry, with the requirement that the electronic energy difference between the S_1_ state at the hopping geometry and S_0_ state equilibrium geometry is partitioned into fundamental modes. Because most of the 87 fundamental modes are spectators in the internal conversion process, only 14 modes gained substantial occupation in the projections. These 14 modes are those that would show HGSC dynamics reflective of the isomerization mechanism.

Initial geometries and velocities for the NAMD trajectories were obtained from ground state sampling at the BH&HLYP/6–31G*level of theory. This involved four ground-state molecular dynamics trajectories that were propagated (≈ 0.48 fs steps) and thermalized with a quantum thermostat^64,65^ using the ABIN code^66^ interfaced to Gaussian 16, initiated from the optimized geometry with velocities obtained from a Boltzmann distribution. The Generalized Langevin Equation (GLE) thermostat parameters (drift and diffusion matrices A and C) were collected from the GLE4MD webpage^67^ for *T* = 298.15 K, along with *N*_s_ = 6 (additional degrees of freedom), ℏω_max_/*k*_B_ *T* = 20, in the strong coupling regime. ω_max_ represents the maximum fundamental mode frequency for which the GLE parameters were optimized, for a particular temperature. At *T* = 298.15 K, ω_max_ = 4114.5 cm^–1^, which is larger than the highest frequency fundamental mode. Equilibration time of the trajectories was determined by monitoring the convergence of the average kinetic energy temperature.^68^ The initial geometries and velocities for NAMD trajectories were obtained by taking snapshots at ≈ 100 fs intervals from the thermalized portion of the thermostat trajectories.

### Experimental Section

Ultrafast TR-IR spectroscopy on cyan (1–2 mM in CD_3_OD, *T* = 293 K) was performed using the ULTRA LifeTime system at the Central Laser Facility, Research Complex at Harwell, Rutherford Appleton Laboratory, UK.^69^ The sample, in a 50 μm path length CaF_2_ cell, was excited with 400 nm light pulses (500 nJ, 150 μm spot size) at a 1 kHz repetition rate, and probed with a delayed IR pulse to capture transient vibrational spectra (cross correlation ≈ 200 fs). The sample position was rasterd in two dimensions during measurements to minimize localized photobleaching and degradation. Measurements were performed with and without the pump pulse (pump-on/pump-off) under magic angle polarization geometry. Spectral calibration used a standard polystyrene IR spectrum.

## Results and Discussion

The computed IR spectrum for (gas phase) cyan, shown in Figure 2a (*T* = 0 K, B3LYP/N07D VPT2 with resonance polyad mixing via Spectro)^52,53^ over the experimental TR-IR window (1350–1850 cm^–1^). Over this window, the intensity arises from 62.8% fundamental modes, 33.8% combination bands (almost exclusively 1 + 1), and 3.4% first overtones. While empirical scaling of harmonic frequencies largely corrects for frequency differences,^70^ it does not remedy inadequate band intensities for fundamental modes from the harmonic approximation. Furthermore, intensity deviations between VPT2 and the resonance polyad corrected spectrum,^71^ stemming from resonance and intensity sharing (notably, the intensity of carbonyl stretch mode ν_14_ is decreased), are sufficiently different to conclude that harmonic intensities are poor. The most pronounced mode, ν_15_, corresponds to methylene bridge C=C stretching, and is flanked by two 1 + 1 combination bands (ν_84_ + ν_10_ and ν_59_ + ν_49_) and the only significant overtone (ν_54_^2^); the summed intensity for these three flanking vibrations exceeds that for ν_15_. Mode ν_19_ is linked with C–C (single bond) stretching on the methylene bridge. A frequency scaling factor of 0.992 was required to best align our calculated anharmonic frequencies with the experimental spectrum in CD_3_OD. The simulated spectrum for *T* = 300 K (gas phase) cyan using VPT2 data (but no resonance polyad mixing via Spectro) is shown in Figure 2b. There is good agreement over the ν_15_ region with the CD_3_OD spectrum, although the ν_14_ (carbonyl stretch) region is particularly difficult to describe due to strong solute–solvent hydrogen bonding.^72^ Furthermore, some extent of deviation across the entire spectrum is expected due to molecular fluxionality at room temperature, the vibrational Stark effect, and other solute–solvent interactions.^72,73^ Unfortunately, cyan is not soluble in nonpolar solvents, which would best approximate the gas-phase case and provide clearest comparison with theory, so we focus on the ν_15_ mode as it is not strongly coupled with solvent. On the other hand, CD_3_OD is a desirable solvent for TR-IR due to an IR-transparent window over the important C=C stretching mode; many nonpolar solvents are not suitable for this frequency range.

**Figure 2:**
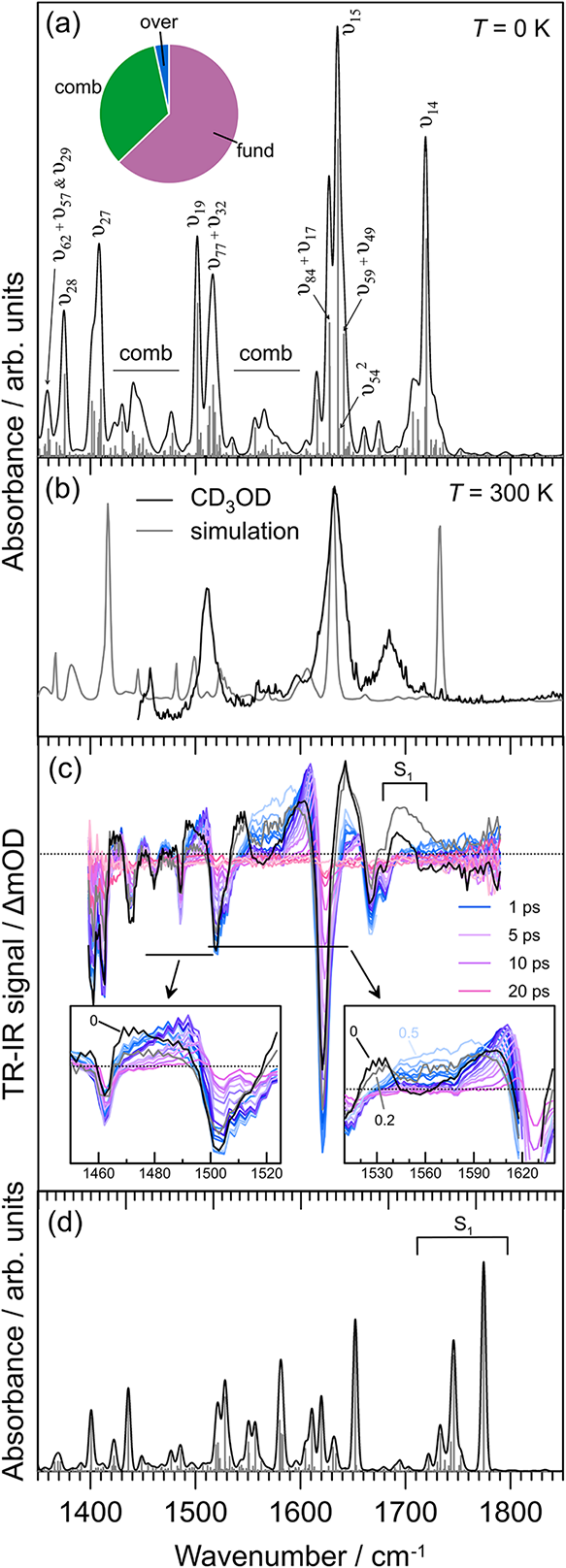
IR and TR-IR spectroscopy of cyan: (a) calculated IR spectrum with 5 cm^–1^ Gaussian convolutions, (b) *T* = 300 K IR spectrum recorded in CD_3_OD and MUCA simulated spectrum, (c) TR-IR data following 400 nm excitation in CD_3_OD (black [0.0 ps] and gray [0.2 ps] show substantial contributions from the S_1_ state), (d) calculated IR spectrum for the S_1_ state (VPT2, BH&HLYP/N07D). Note the blue-shifting over the 1700–1800 cm^–1^ range, which is connected with rapid loss of S_1_ population and corresponding TR-IR signal in (c).

Experimental TR-IR spectra for cyan in CD_3_OD are shown in Figure 2c, showing substantial HGSC band evolution, with the two most important regions highlighted in the inset. It is worth noting that the two shortest pump–probe delays (0 and 0.2 ps) show contributions from IR bands associated with the S_1_ electronic state (Figure 2d).^31^

The NAMD trajectories, used to obtain the initial occupation numbers *n*_*i*_ to model HGSC, are summarized in Figure 3a. They provide the S_1_ → S_0_ decay lifetime of τ_1_ = 535 fs, which is consistent with ultrafast transient absorption measurements at 350 ± 80 fs (ethanol, η = 1.07 cP) and 580 ± 130 fs (ethylene glycol, η = 16.1 cP).^31^ The NAMD *Z* → *E* photoisomerization quantum yield, ϕ_*Z*–*E*_, was 46.4% due to a friction-free environment, and there was no statistical difference in decay lifetime for trajectories forming *E vs Z* isomers. The average time-dependent fluorescence oscillator strength for all trajectories, *f*_osc_ in Figure 3b, provides a similar excited state fluorescence lifetime, and is again consistent with femtosecond fluorescence upconversion lifetimes (which agree well with τ_1_ lifetimes from transient absorption).^31^ The minimum energy crossing point (MECP, Figure 3c) has a twisted methylene bridge (φ_I_ = 85.6°), with the surface hopping geometries showing a moderately tight geometric distribution about the CI geometry, and only a small degree of pyramidization, θ_pyr_, of C(2).^74^ Because ϕ_*Z*–*E*_ in solution is only a few percent,^75^ surface crossings presumably occur mostly for values of φ_I_ less than the MECP value; these crossings are localized in the quadrant containing the star in Figure 3c, left.

**Figure 3:**
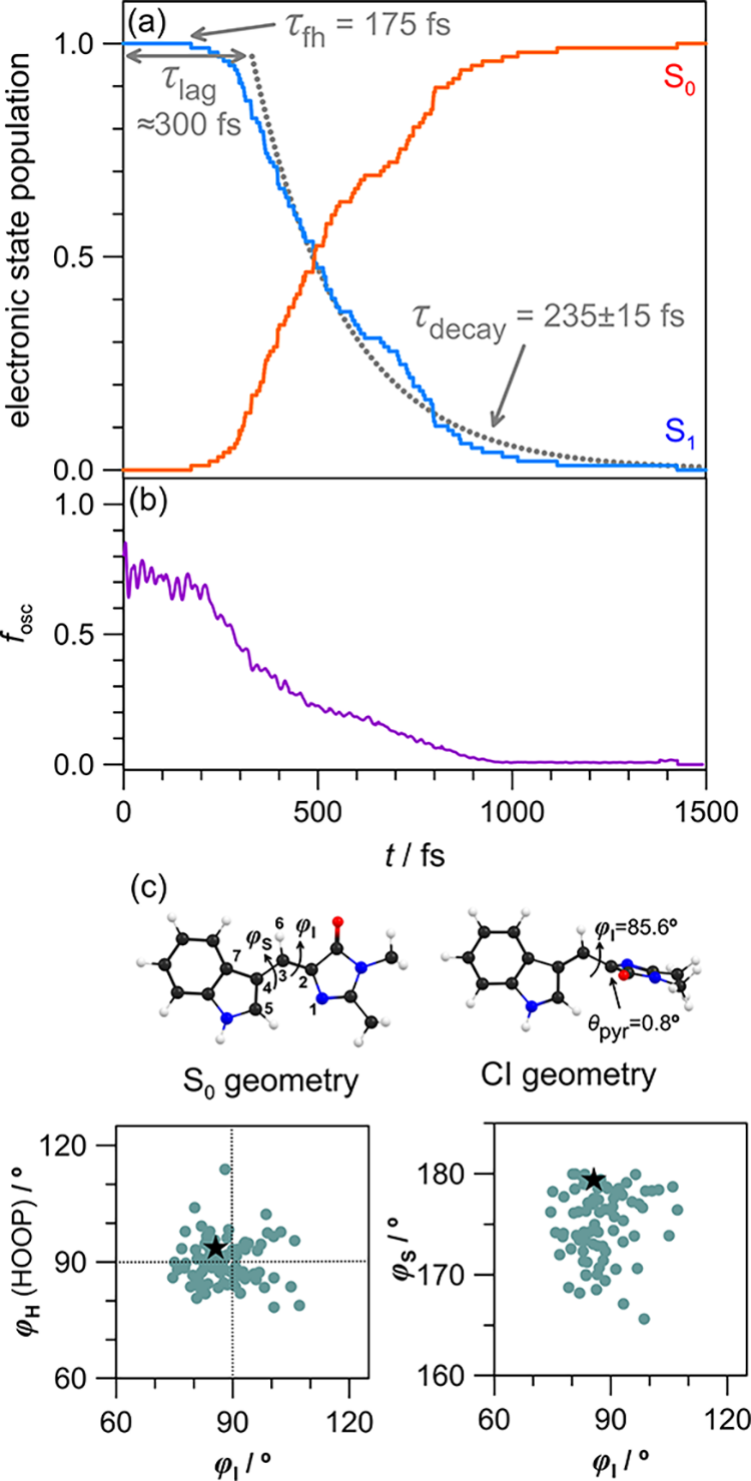
NAMD trajectories for isolated cyan: (a) Evolution of S_1_ and S_0_ state populations with time. With an initial onset region before dihedral twisting (τ_lag_ ≈ 300 fs and first hop τ_fh_ ≈ 175 fs) and an exponential fit to the decay portion of the S_1_ state population (lifetime τ_decay_ = 235 fs), providing the excited state lifetime of τ_1_ = τ_lag_ + τ_decay_ = 535 fs. (b) Evolution of average fluorescence oscillator strength, *f*_osc_. (c) Illustration of the S_0_ state equilibrium and CI geometries, noting dihedral angles φ_I_ (1–2–3–4) and φ_S_ (2–3–4–7). The hydrogen-out-of-plane (HOOP) angle is φ_H_ (6–3–4–7). The pyramidalization angle, θ_pyr_, is defined for the CI geometry. The lower two plots show surface-hopping geometries, with the star representing the optimized minimum energy crossing point (MECP).

The simulated TR-IR spectra for cyan are shown in Figure 4a. There are clear HGSC dynamics over the 1550–1600 cm^–1^ range covering the C=C stretch mode ν_15_, and similar cooling over the 1440–1490 cm^–1^ (ν_19_) range. It is important to note that these cooling dynamics encapsulate the relevant 1 + 1 combination bands and first overtones along with the fundamental bands, so that the HGSC rates always correlate with multiple modes. To compare with experiment, the expectation time-dependent frequency, ⟨ν⟩, was computed over each HGSC band (Figure 4b), revealing good agreement with experiment. On the other hand, HGSC assuming a statistical vibrational distribution in the rapid exchange limit (but no IVR) gave the dashed line in Figure 4b, which deviates from experiment. Similar agreement is found over the ν_15_ mode, although there are several overlapping modes and a nearby 1 + 1 combination band (ν_77_ + ν_32_) that involve heteroatoms that will be strongly coupled with hydrogen-bonding solvents. In addition, HGSC is evident over the 1650–1730 cm^–1^ range, containing the carbonyl stretch mode (ν_14_), where solute–solvent hydrogen bonding effect is strong.

**Figure 4:**
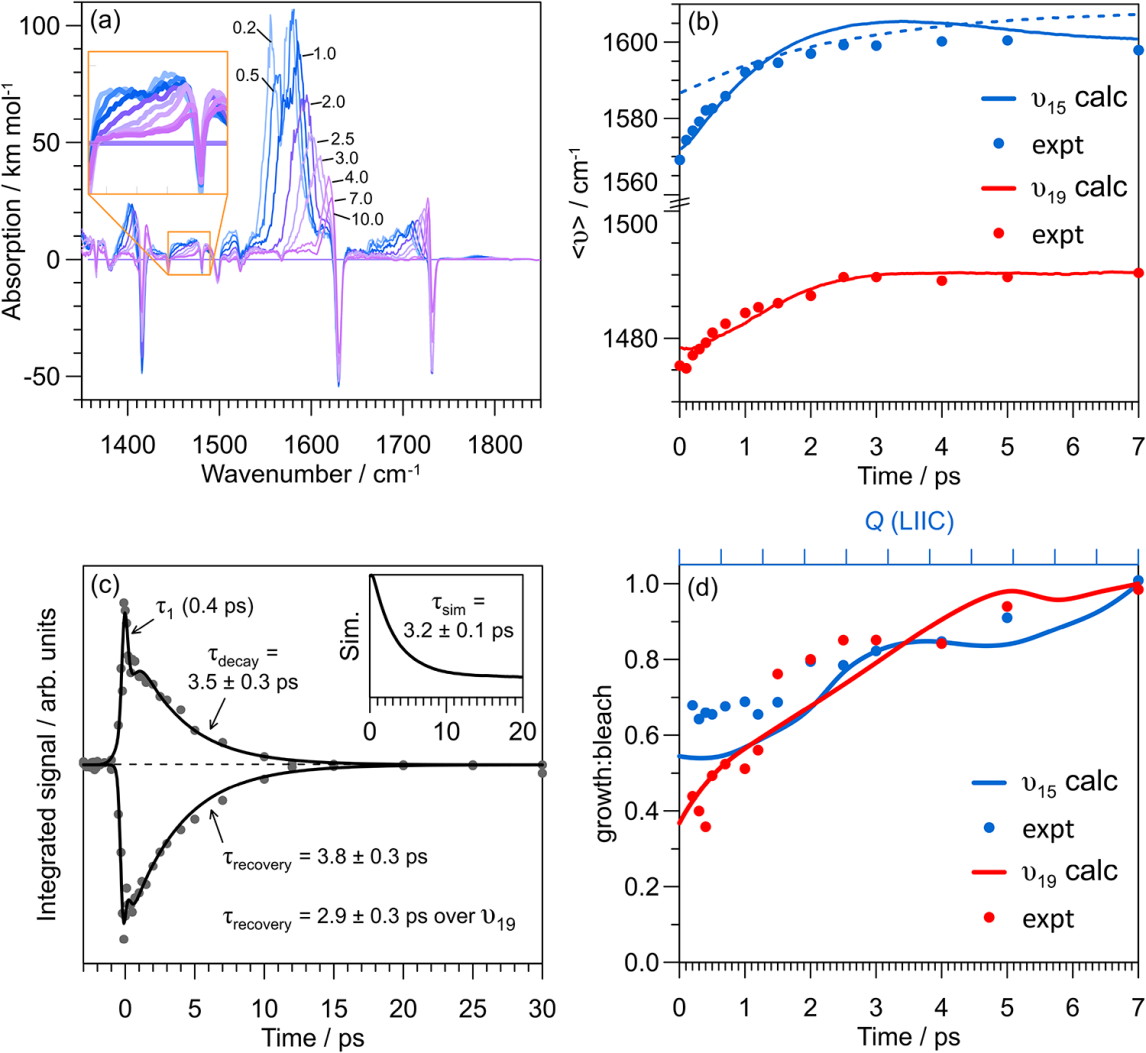
HGSC modeling for cyan: (a) TR-IR spectra over the experimental window modeled using the adjustable parameter *q* = 0.1. The inset shows cooling over ν_19_. (b) HGSC band expectation frequency, ⟨ν⟩, over the two marker bands; this metric quantifies band reshaping. Experimental data are given as points, modeling using *n*_*i*_ from NAMD trajectories are the solid lines, while the dashed line corresponds to an initial statistical distribution. (c) Experimental HGSC lifetimes derived from numerical integration over the strongest TR-IR bands shown in Figure 2c (centered over ν_15_). The inset shows the modeled value over the ν_15_ band assuming the same numerical integration range. (d) Calculated (harmonic) change in *I*, as growth:bleach ratio, for ν_15_ and ν_19_ integration windows with relaxation along the potential energy surface LIIC between the MECP and equilibrium geometry. Experimental data points are taken from numerical integration of growth and depletion bands from insets in Figure 2c.

Fits to the integrated experimental TR-IR absorption and bleach signals over ν_15_ band to a sequential first-order model, $S_{1}\overset{\tau_{1}\;}{\to }S_{0}^{*}\overset{\tau_{2}\;}{\to }S_{0}$ , where τ_2_ is τ_decay_ or τ_recovery_, are shown in Figure 4c. Component τ_1_ is linked with decay of the S_1_ electronic state.^31^ The HGSC lifetimes from TR-IR are longer than those from transient absorption spectroscopy (e.g., τ_2_ = 2.5 ± 0.5 ps in ethanol),^31^ which is expected because the two techniques probe different aspects of HGSC. Specifically, in transient absorption spectroscopy with a concentration *c* of electronic ground state absorbing species

7 $$I_{\mathrm{HGSC}}\propto \left\langle \sigma \left(t\right)\right\rangle c\left(t\right)-\left\langle \sigma_{300\;K}\right\rangle \left[c_{300\;K}-c\left(t\right)\right]$$

where *c*_300 K_ ≫ *c*(*t*). Here, the expectation cross-section for probe absorption ⟨σ­(*t*)⟩, as approximated by vertical excitation oscillator strength along the LIIC,^31^ decreases near the conical intersection geometry. However, it is normal procedure in the transient absorption community to assume an invariant ⟨σ­(*t*)⟩. The implication is that transient absorption spectroscopy is more sensitive toward measuring a subpopulation of the cooling hot ground state ensemble. However, TR-IR better probes the entire HGSC ensemble, but band intensity changes with nuclear coordinate depend on several factors. For example, the simulated HGSC growth feature has more intensity than the corresponding bleach, which contrasts with experiment (Figure 2c). There are several complicating factors that contribute: (i) All trajectories in the HGSC model, using *n*_*i*_ from NAMD trajectories, start at *t* = 0. In the experiment, there will be a distribution of initiation times depending on a convolution of τ_1_ and the pump–probe cross-correlation (≈ 200 fs). The consequence is that experimental HGSC transients will be broadened for early *t*. (ii) TR-IR spectra for photoisomerization reactions can often show more ground-state bleach than corresponding hot ground state absorption (Figure 1b), which is in part due to a changing average dipole moment with molecular distortion from the equilibrium geometry (but still corresponding to the same potential energy surface minimum). Specifically, the absorption intensity, *I*, for a fundamental mode transition has dependence

8 $$I_{n_{i}\to n_{i}+1}\propto {\left|{\left(\frac{\partial \mu }{\partial Q}\right)}_{Q=0}\right|}^{2}$$

where **μ** is the dipole moment vector and *Q* is a nuclear coordinate, so that **μ**(*Q*_0_) is the permanent dipole at the equilibrium geometry. With increasing *Q* along the reaction coordinate away from the equilibrium geometry, the variation in **μ** can be described by the usual expansion

9 $$\mu \left(Q\right)\approx \mu \left(Q_{0}\right)+\mu^{\prime }\left(Q_{0}\right)\left(Q-Q_{0}\right)+\frac{1}{2}\mu^{\prime \prime }\left(Q_{0}\right){\left(Q-Q_{0}\right)}^{2}+...$$

Increasing *Q* on the same potential energy surface shifts the expansion point in eq 9 toward higher terms such that first overtones, 1 + 1 combination bands, and symmetry-broken modes become important leading to intensity changes. While the TR-IR data suggest that the effect is only moderate for cyan (Figure 4d), it may be approximated by computing **μ**(*Q*) and Hessian matrices along *Q*, which involved assuming a linear interpolation (11 points in total) in internal coordinates between the equilibrium and MECP geometries.^31^ The calculated changes for fundamental modes ν_19_ and ν_15_, which are the major HGSC marker modes in the TR-IR measurements (Figure 2c), are shown in Figure 4d and follow the integrated ratios of the experimental data.

The modeling has no explicit treatment of IVR, rather our expression for *p*_down*,i*_ = *qn*_*i*_ν_*i*_ *dt* captures the total HGSC process over the experimental TR-IR window (limited by solvent absorption). In principle, IVR is facilitated by anharmonic couplings with low frequency modes acting as doorway modes for IET. The good agreement found for simulated HGSC properties in this work with experiment is because IVR and IET have similar rates. Ideally, measurement of TR-IR data over an extended spectral window, e.g., down to several hundred wavenumbers (experimentally challenging), and in a series of solvents with differing solvent–solute interaction (e.g., with and without hydrogen bonding character, and also changing viscosity), would allow for clearer consideration of IVR.

A key question is how sensitive/unique are the modeled HGSC spectral signatures? While an answer is molecule-specific, depending on each mode and anharmonic constants, there is generally no strong dependence on *n*_*i*_ when varied by a quantum or so for given mode. This is particularly true when the HGSC transients are complicated by several overlapping modes, such as 1 + 1 combinations bands and fundamentals. Consequently, it is not possible to use experimental HGSC transients to determine details of the photoisomerization mechanism. On the other hand, HGSC modeling would ideally consider an extended spectral range (often limited by the solvent), allowing observation of many active modes, or across multiple solvents. In such a case, analysis should consider also which modes (fundamental and 1 + 1 combination bands) do not show HGSC evolution (i.e., spectator modes) due to low occupation numbers.

## Conclusions

An anharmonic cascade framework for modeling hot ground state cooling in ultrafast photoisomerizing molecules has been reported. This relatively simple model is able to capture properties of HGSC transients for active, marker modes that are not strongly perturbed by solute–solvent interactions. The HGSC framework is readily extendible to similar molecules, such as (barrierless) *Z* stilbenes, ultrafast photoisomerizing retinals, or *E-Z*–type molecular photoswitches. Our demonstration on cyan considered a restricted wavelength window due to solvent absorption, where we were able to observe a diagnostic C=C stretch mode linked with the isomerization process. Spectator modes, which are not involved in isomerization, will not show marked HGSC spectral evolution. Because HGSC transients correspond to an averaged response measurement and many combinations (e.g., initial occupation numbers) could lead to similar spectral outcomes, it is difficult to analyze experimental HGSC transients to infer the dynamics that gave rise to the hot ground state. For systems in which IVR is expected to be substantially more rapid than IET, the HGSC dynamics might be better described in the simpler case involving statistical internal energy distributions.

The HGSC framework applied to cyan used a single adjustable parameter, *q*, linked with the probability for a downward and upward step in *n*_*i*_. In simple terms, the present IET rates capture a decreasing probability with decreasing *n*_*i*_ but neglects all other mode specificity; in the condensed phase, solvents with strong solute–solvent interactions, notably hydrogen bonding, generally facilitate efficient IET. At present this is the main weakness of the approach; a robust treatment for *q* is an exceptionally challenging problem depending on many factors, including solvent modes and solvent–solute mode couplings. The HGSC framework as presented can easily accommodate mode-specific *q*_*i*_, e.g., determined empirically through systematic measurements, removing *q* as an adjustable variable.

## Supplementary Material



## Data Availability

The data that support the findings in this work are available from the corresponding authors upon reasonable request.

## Notes

Tabulation of numerical data for anharmonic modes, illustrations of ν_15_ and ν_19_, and ν_74_ fundamental modes, assignments of fundamental modes, HGSC modeling assuming the rapid exchange limit (PDF)
