# Supplementary material for: Hot Ground State Cooling Following Ultrafast Photoisomerization: Time-Resolved Infrared Spectroscopy
Source: J Phys Chem B. 2025 Dec 10;129(51):13267–76. doi: 10.1021/acs.jpcb.5c07581 (PMC12746466; doi:10.1021/acs.jpcb.5c07581)
Supplement: Supplementary file 1 [file jp5c07581_si_001.pdf]

Supporting Information for 'Hot Ground State Cooling Following Ultrafast Photoisomerization: Time-Resolved Infrared Spectroscopy'

James N. Bull,[*,†] Mark H. Stockett,[*,‡] Pratip Chakraborty,[†] Eleanor K. Ashworth,[†] Anam Fatima,[†] Vincent J. Esposito,[¶] Gregory M. Greetham,[§] Partha Malakar,[§] and Stephen R. Meech[†]

†*Chemistry, Faculty of Science, University of East Anglia, Norwich NR4 7TJ, United Kingdom*

‡*Department of Physics, Stockholm University, SE-10691 Stockholm, Sweden*

¶*Chemistry, Schmid College of Science and Technology, Chapman University, CA 92866, United States*

§*Central Laser Facility, Research Complex at Harwell, Rutherford Appleton Laboratory, Didcot OX11 0QX, United Kingdom*

E-mail: james.bull@uea.ac.uk; mark.stockett@fysik.su.se

First overtone and 1+1 combination band transitions and intensities

The transition energies for the first overtone bands are:

$$\Delta E_{over,i} = 2h\nu_i + 2X_{ii}\left(n_i + \frac{5}{2}\right) + 2\sum_{ij} X_{ij}\left(n_j + \frac{1}{2}\right), \tag{1}$$

and those for the 1+1 combination bands are:

$$\Delta E_{comb,ij} = \Delta E_{fund,i} + \Delta E_{fund,j} + X_{ij}(n_i + n_j + 3). \tag{2}$$

The corresponding intensities are given by:

$$I_{over,i} = I^0_{over,i}\frac{(n_i+1)(n_i+2)}{2}\left(\frac{\Delta E_{over,i}}{\Delta E^0_{over,i}}\right), \tag{3}$$

and

$$I_{comb,ij} = I^0_{comb,ij}(n_i+1)(n_j+1)\left(\frac{\Delta E_{comb,ij}}{\Delta E^0_{comb,ij}}\right). \tag{4}$$

Rapid exchange limit

While IVR is not included in the HGSC model as presented, we can simulate the rapid exchange limit (IVR occurs much more rapidly than IET), where the energized molecules assume a statistical internal energy distribution. The results are shown in Figure S1. Compared with the modeling presented in the paper, there is reduced hot ground state absorption intensity because n_i are much lower for higher frequency modes. Furthermore, contributions from 1+1 combination bands have substantially increased. The simulations assumed q=0.1, although the modeled cooling lifetime has become substantially longer (due to $p_{\mathrm{down},i}$). Changing q can achieve a cooling rate in better agreement with experiment, although does not resolve the poor agreement between $\langle \nu \rangle$ over the ν_{15} mode as presented in the paper.

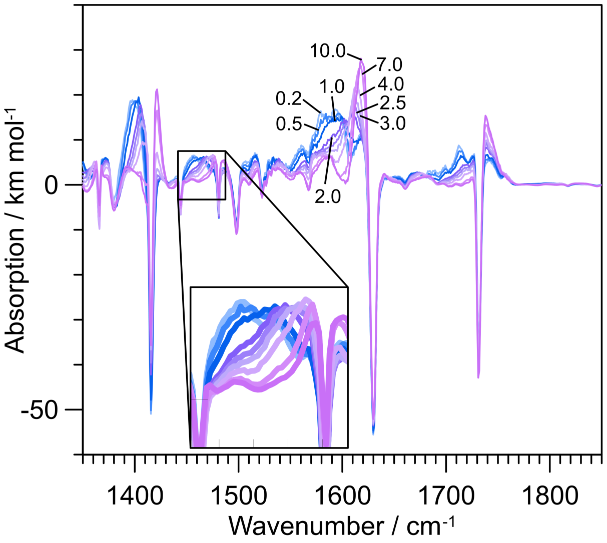

Figure S1: HSGC modeling in the rapid exchange limit with q=0.1. Contributions from fundamental modes have decreased, while 1+1 combination band contributions have increased.

Vibrational modes

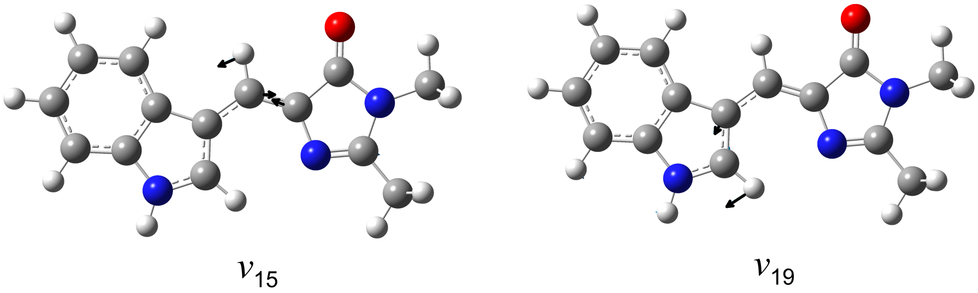

Figure S2: Illustrations of ν_{15} and ν_{19} fundamental modes. Black arrows indicate the displacement vectors.

Illustrations of calculated fundamental modes ν_{15} and ν_{19} for cyan are shown in Figure S2. These are the two main HGSC-active fundamental modes in the experimental TR-IR window. We excluded discussion of the ν_{14} (carbonyl stretch) mode in this work due to strong interaction with solvent.

Mode frequencies, intensities, and assignments for the S_0 and S_1 electronic states are given in Tables S1 and S2, respectively. Assignments follow this atom numbering scheme (S_0 – left & S_1 – right):

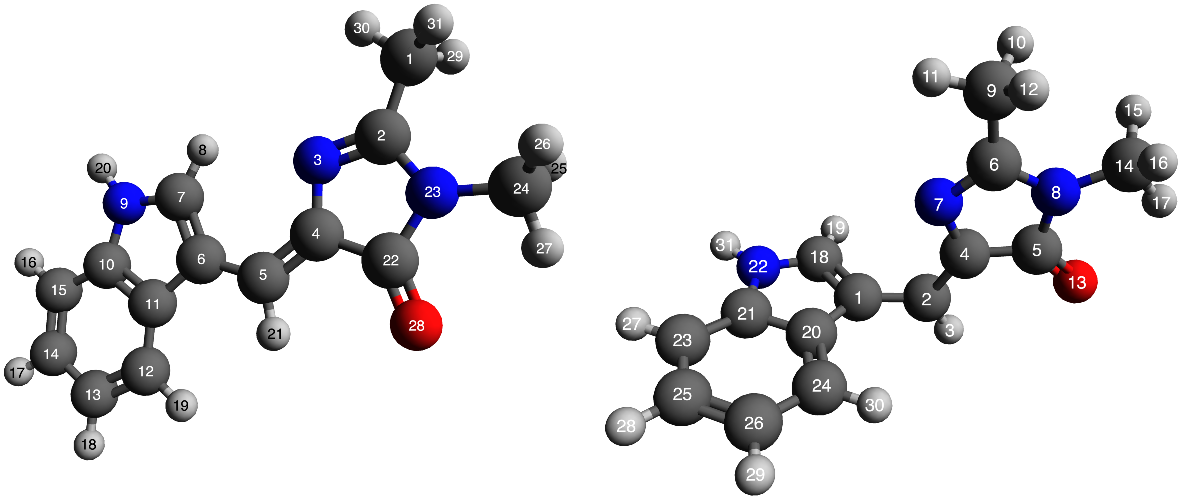

Table S1: Cyan ($Z1$) fundamental frequencies (ν in cm^{-1}), intensities (I in km mol^{-1}), and mode descriptions computed at the B3LYP/N07D level of theory. Subscript 'harm' and 'anh' indicate harmonic and anharmonic values, respectively. The equilibrium geometry has C_S point group symmetry.

Mode	Symmetry	ν_{harm}	ν_{anh}	I_{harm}	I_{anh}	Description
1	a′	3670.5	3506.4	110	88	N(9)H(20) Stretch
2	a′	3287.3	3152.8	9	6	C(7)H(8) stretch
3	a′	3207.7	3070.6	20	17	Phenyl CH symmetric stretch

Continued on next page

Table S1 (continued)

Mode	Symmetry	ν_{harm}	ν_{anh}	I_{harm}	I_{anh}	Description
4	a′	3197.2	3099.9	21	14	Phenyl CH asymmetric stretch
5	a′	3187.3	3042.8	3	4	Phenyl CH asymmetric stretch
6	a′	3181.2	3031.1	2	15	Phenyl CH asymmetric stretch
7	a′	3178.1	3049.4	1	3	C(5)H(21) stretch
8	a′	3158.3	3014.8	5	6	Methyl in-plane C(1)H(30) stretch
9	a′	3143.7	2998.6	1	2	Methyl in-plane C(24)H(27) stretch
10	a″	3098.8	2949.6	23	25	C(24) Methyl out-of-plane asymmetric stretch
11	a″	3089.6	2943.7	6	8	C(1) Methyl out-of-plane asymmetric stretch
12	a′	3040.2	2934.3	100	29	C(1) and C(24) methyl CH symmetric stretch
13	a′	3037.4	2917.7	10	2	C(1) and C(24) methyl CH symmetric stretch
14	a′	1768.6	1735.6	334	19	C(22)O(28) stretch
15	a′	1686.6	1649.0	637	369	C(4)C(5) stretch
16	a′	1662.7	1624.0	5	3	Phenyl in-plane CC ring deformation
17	a′	1622.2	1587.4	21	11	Phenyl in-plane CC ring deformation
18	a′	1611.1	1573.2	37	16	C(2)N(3) stretch
19	a′	1556.2	1516.4	243	64	C(5)C(6) & C(6)C(7) stretch
20	a′	1524.1	1490.7	30	3	Phenyl in-plane CH bend
21	a′	1506.6	1464.3	35	22	C(24) methyl scissor
22	a″	1499.5	1460.2	15	12	C(24) methyl scissor
23	a′	1486.3	1456.0	26	10	Phenyl in-plane CH bend
24	a′	1480.5	1445.5	43	43	C(24) methyl umbrella and C(1) methyl scissor
25	a″	1475.3	1436.5	5	0	C(1) methyl out-of-plane twist
26	a′	1462.3	1422.6	50	9	C(24) methyl umbrella and C(1) methyl scissor
27	a′	1459.0	1422.1	171	114	N(9)H(20) wag
28	a′	1425.7	1388.9	110	120	C(24) and C(1) methyl umbrella
29	a′	1406.0	1375.0	63	35	C(5)H(21) wag
30	a′	1393.5	1360.8	36	42	C(1) methyl umbrella
31	a′	1378.8	1351.2	31	20	Phenyl in-plane CC ring deformation
32	a′	1350.1	1324.0	43	25	Phenyl symmetric in-plane symmetric CH wag

Continued on next page

Table S1 (continued)

Mode	Symmetry	ν_{harm}	ν_{anh}	I_{harm}	I_{anh}	Description
33	a′	1338.2	1306.5	13	2	C(5)H(21) CH in-plane wag
34	a′	1301.0	1271.8	71	47	C(5)H(21) and C(12)H(9) asymmetric in-plane wag
35	a′	1263.2	1241.8	38	46	N(9)H(20) and phenyl symmetric in-plane CH wag
36	a′	1253.5	1227.7	133	26	N(9)H(20) and phenyl asymmetric in-plane CH wag
37	a′	1204.8	1182.3	25	17	C(24) methyl scissor
38	a′	1178.0	1165.7	18	2	Phenyl asymmetric in-plane CH wag
39	a′	1162.8	1136.6	111	58	C(14)H(17)C(15)H(16) in-plane CH bend
40	a″	1147.8	1129.3	1	0	C(24) methyl out-of-plane twist
41	a′	1147.3	1130.1	5	2	Phenyl symmetric in-plane symmetric CH wag
42	a′	1115.1	1096.7	76	37	N(9)H(20) and C(7)H(8) asymmetric in-plane wag
43	a′	1091.5	1074.8	4	7	C(5)H(21) in-plane wag
44	a″	1052.7	1032.5	2	2	C(1) methyl out-of-plane twist
45	a′	1036.1	1021.8	8	3	Phenyl asymmetric ring breathing
46	a′	1034.6	1013.9	14	15	C(1) methyl in-plane CH wag
47	a′	1009.8	990.4	25	8	C(1) methyl in-plane CH wag
48	a″	977.7	962.9	0	0	Phenyl out-of-plane CH bend
49	a″	939.5	928.9	0	2	Phenyl out-of-plane CH bend
50	a′	938.7	919.6	38	24	C(24) and C(1) methyl asymmetric in-plane CH wag
51	a″	938.1	910.1	5	3	C(5)H(21) out-of-plane CH wag
52	a′	884.4	871.3	3	6	Phenyl azo in-plane ring deformation
53	a″	860.5	835.2	9	8	C(7)H(8) out-of-plane wag
54	a″	855.6	845.9	0	0	Phenyl out-of-plane CH bend
55	a′	834.9	822.9	28	9	C(5)H(8) asymmetric breathing
56	a″	775.6	762.6	1	0	Out-of-plane skeletal bend
57	a′	766.1	754.9	32	32	In-plane phenyl and azo symmetric breathing
58	a″	764.9	755.6	9	0	Out-of-plane phenyl and azo symmetric bend
59	a″	751.1	738.8	74	78	Phenyl symmetric out-of-plane CH bend
60	a′	746.5	735.8	6	4	Asymmetric skeletal breathing
61	a′	713.3	698.6	14	8	Diazo asymmetric breathing

Continued on next page

Table S1 (continued)

Mode	Symmetry	ν_{harm}	ν_{anh}	I_{harm}	I_{anh}	Description
62	a″	678.4	668.9	8	7	Diazo out-of-plane bend
63	a″	639.5	627.7	0	0	C(7)H(8) and N(9)H(20) asymmetric out-of-plane wag
64	a′	623.7	616.8	13	12	Skeletal breathing
65	a′	594.9	588.9	11	11	Skeletal breathing
66	a″	583.8	574.4	7	5	Phenyl asymmetric out-of-plane CH bend
67	a′	569.9	564.1	29	28	Skeletal breathing
68	a′	553.6	547.9	20	19	Skeletal breathing
69	a″	505.7	531.3	10	6	Azo and diazo CC, NH, and CH out-of-plane bend
70	a″	476.1	446.5	68	40	N(9)H(20) out-of-plane wag
71	a′	458.0	453.1	16	14	Phenyl azo asymmetric bend
72	a″	429.2	417.8	14	40	Phenyl symmetric CH wag
73	a″	356.7	352.5	2	3	Out-of-plane skeletal bend
74	a′	329.5	325.1	2	2	Methyl carbonyl in-plane wag
75	a′	272.8	272.5	5	3	C(1) and C(24) methyl asymmetric in-plane bend
76	a″	243.8	240.7	7	5	Out-of-plane skeletal bend
77	a″	225.6	220.3	5	4	Phenyl azo symmetric out-of-plane bend
78	a′	224.6	220.7	0	1	Skeletal asymmetric bend
79	a″	206.7	195.6	0	0	Diazo N(23) out-of-plane bend
80	a″	170.7	222.1	0	3	C(1)H(30) out-of-plane wag
81	a′	167.3	161.6	1	1	Asymmetric skeletal bend
82	a″	144.1	41.8	0	2	Dimethyl torsion
83	a″	114.3	140.9	2	1	Symmetric dimethyl torsion
84	a″	104.7	102.7	1	1	Asymmetric dimethyl torsion
85	a′	68.2	57.9	1	1	Symmetric skeletal bend
86	a″	55.6	49.1	3	1	Symmetric skeletal torsion
87	a″	34.9	20.5	1	0	Asymmetric skeletal torsion

Table S2: Cyan ($Z1$) twisted S_1 electronic state fundamental frequencies (ν in cm^{-1}), intensities (I in km mol^{-1}), and mode descriptions computed at the BH&HLYP/N07D level of theory. The equilibrium geometry has C_1 point group symmetry.

Mode	Symmetry	ν_{harm}	ν_{anh}	I_{harm}	I_{anh}	Description
1	a	3765.7	3608.8	137	108	N(22)H(31) stretch
2	a	3381.7	3256.5	8	4	C(18)H(19) stretch
3	a	3330.1	3215.4	6	13	C(24)H(30) stretch
4	a	3315.6	3194.5	6	2	Phenyl symmetric CH stretch
5	a	3308.0	3162.8	19	15	C(2)H(3) stretch
6	a	3304.9	3141.0	6	5	Phenyl asymmetric CH stretch
7	a	3294.5	3187.9	0	0	Phenyl asymmetric CH stretch
8	a	3233.8	3093.1	9	1	Methyl C(9)H(11) stretch
9	a	3232.4	3100.8	2	12	Methyl C(14)H(17) stretch
10	a	3169.7	3032.0	34	32	C(14) methyl asymmetric CH stretch
11	a	3144.3	3001.9	22	27	C(9) methyl asymmetric CH stretch
12	a	3115.1	2965.7	124	4	C(14) methyl symmetric CH stretch
13	a	3101.8	3006.3	61	6	C(9) methyl symmetric CH stretch
14	a	1774.5	1743.3	596	82	C(5)O(13) stretch
15	a	1730.3	1689.8	25	9	Phenyl ring CC stretch
16	a	1694.9	1653.0	27	15	Phenyl ring CC stretch
17	a	1652.2	1610.9	389	140	C(6)N(7) stretch
18	a	1619.9	1581.3	192	95	C(2)C(4) stretch
19	a	1595.2	1555.1	7	4	C(18)N(22) stretch
20	a	1562.7	1530.0	25	6	C(14) methyl scissor
21	a	1557.0	1523.3	109	30	Phenyl azo asymmetric CH bend
22	a	1554.9	1511.5	12	13	C(14) methyl scissor
23	a	1551.0	1496.9	44	8	C(14) methyl umbrella
24	a	1550.3	1519.5	72	64	Phenyl azo symmetric CH bend
25	a	1526.3	1484.1	3	5	C(9) methyl scissor
26	a	1521.5	1476.9	69	44	C(9) methyl scissor
27	a	1507.0	1474.0	17	6	Phenyl azo symmetric CH bend

Continued on next page

Table S2 (continued)

Mode	Symmetry	ν_{harm}	ν_{anh}	I_{harm}	I_{anh}	Description
28	a	1486.0	1448.8	51	34	C(14) and C(9) methyl umbrella
29	a	1454.9	1420.7	17	11	C(9) methyl umbrella
30	a	1436.1	1400.7	190	121	Phenyl azo breathing
31	a	1422.0	1391.4	15	14	Phenyl ring CC stretch
32	a	1371.4	1338.1	20	20	Diazo ring stretch
33	a	1369.1	1346.2	21	10	Phenyl symmetric CH wag
34	a	1343.4	1321.6	59	37	C(2)H(3) in-plane wag
35	a	1274.6	1256.2	10	15	C(1)C(18) stretch
36	a	1246.0	1225.9	39	3	C(14) methyl CH bend
37	a	1242.5	1212.9	31	42	C(14) methyl CH bend
38	a	1213.7	1197.3	2	0	C(2)H(3) in-plane wag and phenyl asymmetric CH bend
39	a	1209.4	1185.4	0	1	C(2)H(3) in-plane wag and phenyl asymmetric CH bend
40	a	1192.7	1170.3	1	0	C(14) methyl CH scissor
41	a	1189.2	1172.0	26	11	C(18)H(19) and N(22)H(31) asymmetric bend
42	a	1153.2	1134.6	1	1	Phenyl asymmetric CH bend
43	a	1111.1	1097.8	17	4	C(2)H(3) bend
44	a	1085.2	1058.2	1	0	C(9) methyl twist
45	a	1079.7	1057.0	21	3	Dimethyl CH rocking
46	a	1065.6	1051.4	1	7	Phenyl asymmetric breathing
47	a	1050.5	1037.1	0	0	Phenyl out-of-plane asymmetric CH bend
48	a	1041.1	1022.7	13	10	Diazo methyl ring deformation
49	a	1018.6	1000.4	1	2	Phenyl out-of-plane asymmetric CH bend
50	a	966.4	957.5	1	3	Dimethyl CH rocking
51	a	939.6	915.5	4	7	C(18)H(19) out-of-plane wag
52	a	926.6	910.9	2	0	Phenyl out-of-plane asymmetric CH bend
53	a	903.0	892.5	3	4	Phenyl azo ring stretch
54	a	850.5	835.7	13	7	Skeletal deformation
55	a	803.9	802.6	46	5	Phenyl out-of-plane symmetric CH bend
56	a	802.1	782.9	20	21	Phenyl out-of-plane symmetric CH bend

Continued on next page

Table S2 (continued)

Mode	Symmetry	ν_{harm}	ν_{anh}	I_{harm}	I_{anh}	Description
57	a	782.9	770.2	10	44	Phenyl out-of-plane symmetric CH bend
58	a	775.8	758.5	1	1	C(5)C(4)N(7) out-of-plane bend
59	a	766.7	756.4	2	2	Skeletal deformation
60	a	738.6	727.4	8	13	Skeletal deformation
61	a	677.9	664.5	7	10	Diazo methyl out-of-plane bend
62	a	652.0	640.2	37	26	N(22)H(31) out-of-plane wag
63	a	640.3	633.7	11	13	C(6)C(9) stretch
64	a	617.6	610.3	8	5	Azo ring breathing
65	a	593.1	582.4	10	15	Phenyl azo out-of-plane bend
66	a	592.2	587.6	6	7	N(22)H(31) out-of-plane bend
67	a	580.1	567.0	15	6	N(22)H(31) out-of-plane bend
68	a	556.5	531.0	52	50	NH(31) and C(18)H(19) symmetric out-of-plane bend
69	a	518.8	509.2	2	2	Phenyl azo asymmetric bend
70	a	468.9	476.6	31	35	C(2)H(3) out-of-plane wag
71	a	431.4	429.6	6	15	Phenyl azo out-of-plane symmetric bend
72	a	395.9	412.1	27	10	Skeletal deformation
73	a	355.1	351.4	8	1	Skeletal deformation
74	a	325.3	321.9	4	4	C(14) methyl carbonyl symmetric bend
75	a	301.7	306.3	3	1	C(2) out-of-plane bend
76	a	274.3	267.8	5	2	Dimethyl asymmetric bend
77	a	230.3	213.4	11	16	Skeletal deformation
78	a	224.0	240.4	7	5	Skeletal deformation
79	a	208.7	199.6	11	9	Skeletal breathing
80	a	177.9	183.8	1	4	C(9) methyl torsion
81	a	139.5	298.8	0	3	Dimethyl torsion
82	a	137.4	132.5	2	3	Skeletal torsion
83	a	122.5	44.5	6	1	Dimethyl torsion
84	a	100.6	16.6	1	2	C(14) methyl torsion
85	a	42.4	12.0	4	1	Skeletal rocking

Continued on next page

Table S2 (continued)

Mode	Symmetry	ν_{harm}	ν_{anh}	I_{harm}	I_{anh}	Description
86	a	31.5	0.5	2	0	Skeletal rocking
87	a	15.9	1.0	1	0	Skeletal rocking
